# A case for South-South collaboration for trachoma elimination

**Published:** 2017-02-10

**Authors:** Mwele Malecela, Upendo Mwingira, Sultani Matendechero, Michael Gichangi, Rebecca Oenga, Paul Emerson, Teshome Gebre, Girija Sankar

The East Africa NTD/Trachoma Cross-Border Partnership brings together representatives from the same ‘neighbourhood’ – Eritrea, Ethiopia, Kenya, South Sudan, Sudan, Tanzania and Uganda – to share experiences of common interest in the delivery of trachoma and other neglected tropical disease (NTD) programmes. These countries understand that they will never reach their individual elimination targets without working together: they are all home to nomadic populations of pastoralists who live on both sides of an international border and are bound more closely by relations, socio-cultural activities and trade than by borders. There are also common programmatic challenges because of shared histories, ethnicities, and languages, an understanding and appreciation of which are critical to provide effective public health services.

The Ministry of Health, Community Development, Gender, Elderly and Children in Tanzania hosted the second annual meeting of this partnership in August 2016. The discussion fostered by the three days of meetings were inspirational, educational and led to concrete actions that will accelerate progress towards the elimination of blinding trachoma and other NTDs.

## Finding ways forward

One of the highlights was the first meeting of the district officials with responsibility for implementing the programmes for Maasai communities in Kenya and Tanzania. They were able to share their successes and challenges in working with the Maasai, leading to several ‘lightbulb’ moments of greater understanding. Likewise, representatives from Ethiopia, Kenya, South Sudan and Uganda (home to the Ateker people, comprising the Jie, Karamajong, Nyangatom, Turkana, and Toposa tribal groups) identified areas for collaborative engagements in NTD and trachoma service provision along the Ateker corridor, including coordinating surgical services and sharing Ateker-speaking surgeons. The Galabat East district in Sudan and the Metema district in Ethiopia have reached their trachoma elimination targets and plans are now in place for joint surveillance activities on either side of the border.

**Figure:**
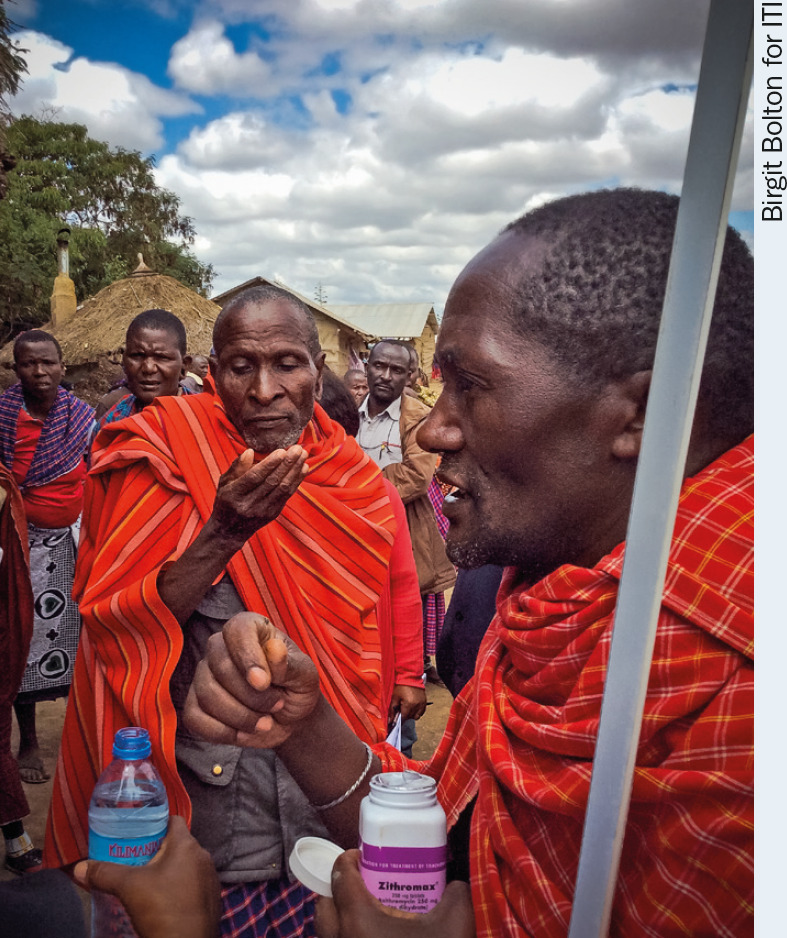
Mass drug administration (MDA) amongst Maasai communities in Monduli district. TANZANIA

Countries also planned ways to synchronise mass drug administration activities, share health education materials, assist in human resource development (where gaps were identified), enhance efforts on facial and environmental hygiene in villages and schools along the border, and collaborate on surveys.

Global alliances of NGOs and donors can offer technical and financial resources but it is the country programmes that are the engines of disease elimination. The programme staff best able to understand the problems and identify solutions for their local contexts are those who work in close proximity with the communities they serve on a day-to-day basis. However, when the policies they are implementing are not working, the next best place to look for solutions is an adjacent district where different solutions may have been developed for a similar set of problems. Global alliances can provide the framework for such knowledge sharing, but it is when district officials adapt (and extend) these frameworks that the success of service delivery is evident. For example, a few weeks after the Arusha meeting, district health officials from Longido, Tanzania and Kajiado, Kenya met in a border town to finalise a coordinated work plan to provide services for the Maasai population on both sides of the border. During the meeting, representatives of the Tanzania programme, which was struggling to gain acceptance and traction in the Maasai communities, was inspired by the experience of the Kenyans, who had spent more time gaining the trust of the Masaai communities, resulting in them becoming partners in the programme and actively seeking out trachoma treatment and surgical services. Similar meetings are planned for the countries along the Ateker corridor.

**“The East Africa partnership is proving to be an essential framework”**

## A case for regional networks

The East Africa partnership is proving to be an essential framework for supporting this group of national programmes, and is a model that should be replicated wherever there are similar groupings of countries that share common issues. For example, countries in Southern Africa, comprising Malawi, Mozambique, Zambia, and Zimbabwe, will benefit from emulating this model because they share some common ethnicities and languages. The islands of the South Pacific-Fiji, Kiribati, Papua New Guinea, Solomon Islands, and Vanuatu – share common operational issues and can benefit from a regional knowledge-sharing network. The partnerships can go far beyond knowledge sharing and include practical solutions such as the sharing of surgeons that speak the same language. They can also enhance efficiency by minimising replication and providing a platform for district teams to learn and benefit from each other's strengths to improve programmes.

National NTD programmes have to be able to see what is possible and learn from their successes and failures, as well as those of their neighbours, to plan and deliver effective services. Similar cross-border collaborations have recently been reported in the onchocerciasis control programmes in the Mano River Union (West Africa) with very similar findings and recommendations.

With unprecedented resource mobilisation for NTDs, it is now hard to describe these diseases of neglected people as themselves neglected. For the resources to be best utilised, however, delivery programmes must be efficient and effective. Sharing experiences can save country programmes years of trial and error and improve access to freedom from disease for all.

